# Generation and characterization of two induced pluripotent stem cell lines (ICGi052-A and ICGi052-B) from a patient with frontotemporal dementia with parkinsonism-17 associated with the pathological variant c.2013T>G in the MAPT gene

**DOI:** 10.18699/vjgb-24-76

**Published:** 2024-11

**Authors:** E.V. Grigor’eva, A.A. Malakhova, E.S. Yarkova, J.M. Minina, Y.V. Vyatkin, J.A. Nadtochy, E.A. Khabarova, J.A. Rzaev, S.P. Medvedev, S.M. Zakian

**Affiliations:** Institute of Cytology and Genetics of the Siberian Branch of the Russian Academy of Sciences, Novosibirsk, Russia Institute of Chemical Biology and Fundamental Medicine of the Siberian Branch of the Russian Academy of Sciences, Novosibirsk, Russia; Institute of Cytology and Genetics of the Siberian Branch of the Russian Academy of Sciences, Novosibirsk, Russia Institute of Chemical Biology and Fundamental Medicine of the Siberian Branch of the Russian Academy of Sciences, Novosibirsk, Russia; Institute of Cytology and Genetics of the Siberian Branch of the Russian Academy of Sciences, Novosibirsk, Russia Novosibirsk State University, Novosibirsk, Russia; Institute of Cytology and Genetics of the Siberian Branch of the Russian Academy of Sciences, Novosibirsk, Russia; NOVEL Ltd., Novosibirsk, Russia; Institute of Cytology and Genetics of the Siberian Branch of the Russian Academy of Sciences, Novosibirsk, Russia Novosibirsk State University, Novosibirsk, Russia; Institute of Cytology and Genetics of the Siberian Branch of the Russian Academy of Sciences, Novosibirsk, Russia Federal Neurosurgical Center of the Ministry of Health of the Russian Federation, Novosibirsk, Russia; Federal Neurosurgical Center of the Ministry of Health of the Russian Federation, Novosibirsk, Russia; Institute of Cytology and Genetics of the Siberian Branch of the Russian Academy of Sciences, Novosibirsk, Russia Institute of Chemical Biology and Fundamental Medicine of the Siberian Branch of the Russian Academy of Sciences, Novosibirsk, Russia; Institute of Cytology and Genetics of the Siberian Branch of the Russian Academy of Sciences, Novosibirsk, Russia Institute of Chemical Biology and Fundamental Medicine of the Siberian Branch of the Russian Academy of Sciences, Novosibirsk, Russia

**Keywords:** frontotemporal dementia with parkinsonism-17; ;, induced pluripotent stem cells, MAPT gene, лобно-височная деменция с паркинсонизмом-17, индуцированные плюрипотентные стволовые клетки, ген MAPT

## Abstract

Frontotemporal dementia with parkinsonism-17 is a neurodegenerative disease characterised by pathological aggregation of the tau protein with the formation of neurofibrillary tangles and subsequent neuronal death. The inherited form of frontotemporal dementia can be caused by mutations in several genes, including the MAPT gene on chromosome 17, which encodes the tau protein. As there are currently no medically approved treatments for frontotemporal dementia, there is an urgent need for research using in vitro cell models to understand the molecular genetic mechanisms that lead to the development of the disease, to identify targets for therapeutic intervention and to test potential drugs to prevent neuronal death. Analysis of exome sequencing data from a 46-year-old patient with a clinical diagnosis of Parkinson’s disease revealed the presence of the pathological variant c.2013T>G (rs63750756) in the MAPT gene, which is associated with frontotemporal dementia with parkinsonism-17. By reprogramming the patient’s peripheral blood mononuclear cells, we obtained induced pluripotent stem cells (iPSCs). Two iPSC lines were characterised in detail. Reprogramming was performed by transfection with non-integrating episomal vectors expressing the OCT4, SOX2, KLF4, LIN28, L-MYC and mp53DD proteins. The iPSC lines ICGi052-A and ICGi052-B proliferate stably, form colonies with a morphology characteristic of human pluripotent cells, have a normal diploid karyotype (46,XX), express endogenous alkaline phosphatase and pluripotency markers (OCT4, NANOG, SSEA-4 and TRA-1-60) and are able to differentiate into derivatives of three germ layers: ento-, ecto- and mesoderm. The iPSC lines obtained and characterised in detail in this work represent a unique tool for studying the molecular genetic mechanisms of the pathogenesis of frontotemporal dementia with parkinsonism-17, as well as for testing potential drugs in vitro.

## Abstract (ru)

Лобно-височная деменция с паркинсонизмом-17 – нейродегенеративное заболевание, характеризующееся патологической агрегацией белка тау с образованием нейрофибриллярных клубков и дальнейшей гибелью нейронов. Наследственная форма лобно-височной деменции может быть вызвана мутациями в различных генах, одним из которых является ген MAPT на хромосоме 17, кодирующий тау-белок. Поскольку на данный момент отсутствуют утвержденные медицинским сообществом способы борьбы с лобно-височной деменцией, исследование на клеточных моделях in vitro молекулярно-генетических механизмов, приводящих к развитию заболевания, поиск мишеней для терапевтического воздействия и возможность тестирования потенциальных лекарственных препаратов для предотвращения гибели нейронов являются актуальной задачей. Анализ данных секвенирования экзома 46-летней пациентки с клиническим диагнозом болезнь Паркинсона показал наличие патологического варианта c.2013T>G (rs63750756) в гене MAPT, который ассоциирован с лобно-височной деменцией с паркинсонизмом-17. При помощи репрограммирования мононуклеарных клеток периферической крови пациентки нами были получены десять линий индуцированных плюрипотентных стволовых клеток (ИПСК), из которых детально охарактеризованы две. Репрограммирование проводили с помощью трансфекции неинтегрирующимися эписомными векторами, которые экспрессируют белки OCT4, SOX2, KLF4, LIN28, L-MYC и mp53DD. Линии ИПСК ICGi052-A и ICGi052-B стабильно пролиферируют, образуют колонии с характерной для плюрипотентных клеток человека морфологией, имеют нормальный диплоидный кариотип (46,XX), экспрессируют эндогенную щелочную фосфатазу и маркеры плюрипотентности (OCT4, NANOG, SSEA-4 и TRA-1-60) и способны дифференцироваться в производные трех зародышевых листков: энто-, экто- и мезодерму. Благодаря тому, что ИПСК можно направленно дифференцировать в широкий спектр типов клеток, полученные в данной работе и детально охарактеризованные линии ИПСК являются уникальным инструментом для изучения молекулярно-генетических механизмов патогенеза лобно-височной деменции с паркинсонизмом-17, а также тестирования потенциальных лекарственных препаратов in vitro.

## Introduction

Frontotemporal dementia with parkinsonism linked to chromosome 17 was first described by T. Lynch and colleagues in 1994 (Lynch et al., 1994). This inherited early-onset dementia syndrome also includes parkinsonism, a set of symptoms that includes both non-motor manifestations (such as cognitive impairment, depression, bipolar disorder, sleep disturbances) and motor disorders such as muscle rigidity, resting tremor, bradykinesia. Early mental disorders and personality changes often precede severe dementia.

The MAPT gene encodes the tau protein, which regulates the assembly and stabilisation of microtubules involved in central nervous system signalling and axonal transport (Esmaeli-Azad et al., 1994). Six isoforms of the tau protein are expressed in the human brain due to alternative splicing of exons 2, 3 and 10 of the mRNA of the MAPT gene. Alternative splicing of exon 10 results in a tau protein with three (3R) or four (4R) microtubule-binding (MT-binding) domains. The 4R tau isoforms have an increased affinity for microtubules compared to the 3R isoforms. In childhood, the 3R form predominates in the brain, whereas in the adult brain, the 3R and 4R forms are present in a 1:1 ratio (D’Souza, Schellenberg, 2006).

The MAPT:c.2013T>G genetic variant (rs63750756, p.N279K) is a missense mutation in exon 10 resulting in the substitution of asparagine for lysine at position 279 of the MAPT protein (Hasegawa et al., 1999). As a result of this substitution, the polypurine-positive cis-element present in exon 10 is enhanced, leading to an increase in the frequency of inclusion of exon 10 in the transcript during splicing (Ritter et al., 2018). This results in a change in the ratio of 3R and 4R forms, leading to microtubule destabilisation, disruption of vesicle intracellular trafficking (Wren et al., 2015), formation of filamentous inclusions (Ghetti et al., 2015) and subsequent neuronal death. In addition, neurons with this genetic variant are characterised by increased spontaneous calcium fluctuations, mitochondrial dysfunction and increased production of reactive oxygen species, which also leads to cell death (Korn et al., 2023).

These processes lead to the development of frontotemporal dementia with parkinsonism-17. The exact mechanism of disease development in this genetic variant is not yet known, making it impossible to develop a treatment regimen.

Several models are used to study the development of frontotemporal dementia. The first are animal models, mainly in rodents: laboratory mice and rats. Rodents are actively used to create models of neurodegenerative diseases (Britti et al., 2020; Esteras et al., 2021). There are also transgenic lines of laboratory animals carrying genetic variants that cause hereditary frontotemporal dementia (Dawson et al., 2007). However, these model systems have some limitations due to differences in the signs of ageing between mice and humans and even differences between mouse and human tau protein (Iovino et al., 2015; Hernández et al., 2020). Therefore, in addition to animal models, models based on induced pluripotent stem cells (iPSCs) are now the most promising

iPSCs are cells obtained by reprogramming somatic cells and are characterised by the ability to differentiate into derivatives of three germ layers like embryonic stem cells. iPSCs have all the properties of embryonic stem cells and are at the same time autologous with respect to the somatic cell donor (Valetdinova et al., 2021). iPSCs have great potential for personalised medicine, as they can be derived at any time in a patient’s life and will be patient-specific. iPSCs make it possible to circumvent immunogenic and ethical issues, so the demand for them is growing. In principle, iPSCs can be generated by reprogramming any mature cell type isolated from the body. Fibroblasts, mononuclear blood cells, keratinocytes and melanocytes are widely used

Because iPSCs can be differentiated into any cell type, their range of applications is very broad (Liu et al., 2020). Differentiated iPSC derivatives, such as neuron-like cells, can be used to study the molecular genetic mechanisms of the development of neurodegenerative diseases, including during the early stages of cell differentiation, to search for target molecules, and to test various chemical compounds as potential drugs (Grigor’eva et al., 2023).

In this work, we have obtained, characterised and certified two lines of iPSCs from a patient with a pathological genetic variant of MAPT:c.2013T>G (rs63750756, p.N279K), which leads to the development of frontotemporal dementia with parkinsonism-17. The resulting iPSC lines provide a cellular model to study the mechanisms of development of this disease and can also be used for drug screening.

## Materials and methods

Compliance with ethical standards. The study was approved by the Research Ethics Committee of the Federal Neurosurgical Center (Novosibirsk, Russia), Protocol No. 1 dated 14 March 2017. Peripheral blood samples of the patient were provided by the Federal Neurosurgical Center (Novosibirsk, Russia). The patient signed a voluntary informed consent and an information sheet.

Isolation of PBMCs using a ficoll gradient. Peripheral blood was collected in two 9 ml vacuum tubes containing K3EDTA (VACUETTE, Greiner Bio). To isolate PBMCs, 3–4 ml of blood was layered on 3–4 ml of ficoll solution (Histopaque-1077, Sigma-Aldrich or Biolot, density 1.077) and centrifuged at 400 g for 35–40 min on a centrifuge with slow rotor acceleration and deceleration (SL 16 Cenrtifuge, Thermo Fisher Scientific). A whitish interphase layer containing PBMCs was then carefully collected, transferred to a 15 ml tube and washed twice in the maximum volume of PBS by centrifugation at 300 g for 15–20 min. PBMCs were frozen in medium containing 90 % KnockOut Serum substitute (Thermo Fisher Scientific) and 10 % DMSO (Sigma-Aldrich), 5–10 million cells per cryovial.

Reprogramming of patient-specific PBMCs and conditions for culturing iPSCs. PBMC transfection was performed on a Neon Transfection System (Thermo Fisher Scientific) device using episomal vectors encoding OCT4, SOX2, KLF4, L-MYC, LIN28, mp53DD and EBNA1 (Addgene ID No. 41813-14, No. 41855-57), as previously described (Grigor’eva et al., 2023). 0.5 micrograms of each vector was used for transfection of 1 × 106 PBMCs, using the programme 1650 V, 10 ms, 3 pulses. Cells were plated on a feeder layer of mitomycin C (Sigma-Aldrich)-treated embryonic fibroblasts. Single cell colonies were manually plated into 1 cm2 wells with a pre-plated feeder and cultured in iPSC medium containing DMEM/F12, 15 % KnockOut Serum Replacement, 1 % GlutaMAX-I, 0.1 mM NEAA, 1 % penicillin-streptomycin (all Thermo Fisher Scientific), 0.1 mM 2-mercaptoethanol (Sigma-Aldrich) and 10 ng/ml bFGF (SCI Store). Cell colonies were enzymatically transplanted every 4–5 days using TrypLE Express (Thermo Fisher Scientific) at a ratio of 1:8–1:10 with the addition of 2 μg/ml ROCK inhibitor Thiazovivin (Sigma- Aldrich) for one day. All cells were cultured in a CO2 incubator at 37 °C in a humid atmosphere with 5 % CO2.

Detection of endogenous alkaline phosphatase. Cells were fixed by air drying and stained with SIGMAFAST BCIP/ NBT reagent (Sigma-Aldrich) according to the manufacturer’s protocol for 10–15 minutes in the dark at room temperature (RT). The preparations were washed with PBS and visualised using a Nikon Eclipse Ti-E microscope (Nikon).

Immunofluorescence staining. For immunofluorescence analysis, cells were placed on 8-well chambered coverslips (Thermo Fisher Scientific), fixed in 4 % paraformaldehyde (PFA, Sigma-Aldrich) for 10 minutes at RT, permeabilized with 0.5 % Triton-X 100 (Sigma-Aldrich) for 30 minutes at RT, and incubated with 1 % BSA (VWR) for 30 minutes at RT. Cells were incubated with primary antibodies overnight at 4 °C, secondary antibodies were added for 1.5 hours at RT (Table 1). The nucleus was counterstained with DAPI. Images were captured using a Nikon Eclipse Ti-E microscope and NIS Elements Advanced Research software version 4.30.

**Table 1.**
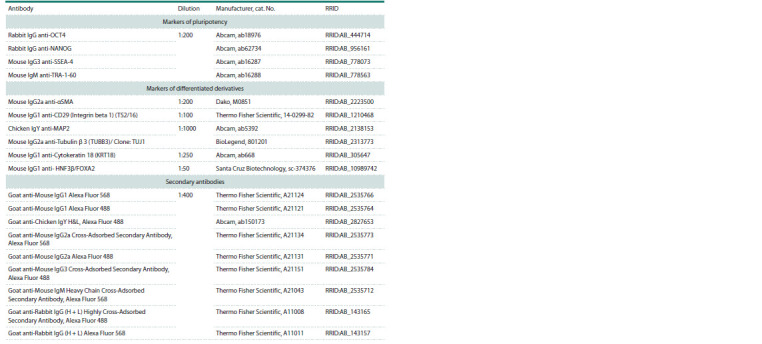
Antibodies used in the work

Spontaneous differentiation of iPSCs in embryoid bodies. In order to determine the potential of the cells to produce three germ layers, spontaneous differentiation of iPSCs through embryoid body formation was performed as previously described (Grigor’eva et al., 2023). Briefly, cells were detached with 0.15 % collagenase type IV (Thermo Fisher Scientific) and plated on 1 % agarose-coated dishes in iPSC culture medium without the addition of bFGF. After 9–14 days, the embryoid bodies were transferred to 8-well Chambered Coverglass Matrigel-coated plates (Thermo Fisher Scientific) and cultured for a further 7–9 days. They were then fixed in 4 % PFA and subjected to immunofluorescence staining. The list of antibodies is shown in Table 1.

Karyotyping of iPSC lines. Cells were expanded to a monolayer and plated on four wells of a 12-well tablet coated with Matrigel (Corning) extracellular matrix proteins and cultured for 48–72 hours, depending on the rate of cell proliferation. 2.5 hours before fixation, the medium was changed to fresh medium, 3 μg/ml ethidium bromide and 50 ng/ml colcemide were added and the cells were left in a CO2 incubator at 37 °C. The cells in the wells were then disaggregated with 300 μl TrypLE Express and 3 ml of a hypotonic solution of 0.28 % KCl were added for 20 minutes at 37 °C. After incubation, 2 drops of Carnois fixative (3 parts methanol, 1 part glacial acetic acid) were added, the cell suspension was carefully transferred to centrifuge tubes and centrifuged at 1,300 rpm for 7 minutes. The cells were fixed by adding 1.5 ml fresh Carnois fixative to the cell pellet for 15 minutes on ice. The cells were then centrifuged for 5 minutes at 1,300 rpm, the Carnois fixative was changed twice and 70–80 μl of the cell suspension was pipetted onto cooled wet slides from a height of 10–20 cm. The preparations were allowed to dry at room temperature.

For differential chromosome staining, the preparations were stained with DAPI solution (200 ng/ml, in 2xSSC) for 5 minutes. The preparations were then rinsed in a 2xSSC buffer and in water. After drying the preparations in air, 7–10 μl of antifade (Vector) was applied under a cover glass.

Chromosome analysis was performed under an Axioplan 2 microscope (Zeiss) equipped with a CV-M 300 CCD camera (JAI Corp.) at the Common Use Center of Microscopy of Biological Objects (https://ckp.icgen.ru/ckpmabo) at the Institute of Cytology and Genetics SB RAS. ISIS 5 software (MetaSystems Group) was used to process the metaphases.

Isolation of genomic DNA and RNA. Genomic DNA was isolated from PBMCs and iPSCs using the DNeasy Blood & Tissue Kit (Qiagen) or by extraction using QuickExtract DNA Extraction Solution (Lucigen). RNA was isolated using Trizol (Thermo Fisher Scientific) according to the manufacturer’s protocol.

Identification of a pathological genetic variant in patient- specific PBMCs and obtained iPSCs. Clinical exome sequencing was performed at Genoanalytica LLC, Moscow (https://www.genoanalytica.ru), using a DNA sample from patient-specific PBMCs. Genomic DNA was ultrasonically fragmented to an average size of 300 nucleotides using the Covaris S2 instrument. After concentration measurement, 800 ng of DNA was used to prepare libraries using the NEBNext® Ultra™ II DNA Library Prep Kit for Illumina (New England Biolabs) according to the manufacturer’s instructions. The resulting library was then hybridised with probes corresponding to protein-coding parts of the human genome using the Sure Select AllExome V7 Kit (Agilent) according to the instructions. After hybridisation, the library was sequenced using paired-end reads of 150 nucleotides on the HiSeq 2500 (Illumina). The raw sequencing data of the PD57 patient exome are available in the SRA database (project PRJNA563295, sample SAMN42050731, https://www.ncbi. nlm.nih.gov/biosample/42050731).

To confirm the presence of the single nucleotide polymorphism, the PCR products were Sanger sequenced using the primers listed in Table 2. The PCR was performed on a T100 thermal cycler (Bio-Rad) using HS-Taq PCR-Color (2×) BioMaster (Biolabmix) and the following programme: 95 °C – 3 min; 35 cycles: 95 °C – 30 s; 68 °C – 30 s; 72 °C – 30 s; and 72 °C – 5 min. The PCR product was purified by electrophoresis in agarose gel followed by separation using the Cleanup Mini Kit (Eurogene) according to the manufacturer’s protocol. Sanger sequencing reactions were performed using the Big Dye Terminator V. 3.1. Cycle Sequencing Kit (Applied Biosystems) and analysed on the ABI 3130XL Genetic Analyser at the Genomics Core Facility SB RAS (http://www. niboch.nsc.ru/doku.php/corefacility ).

**Table 2.**
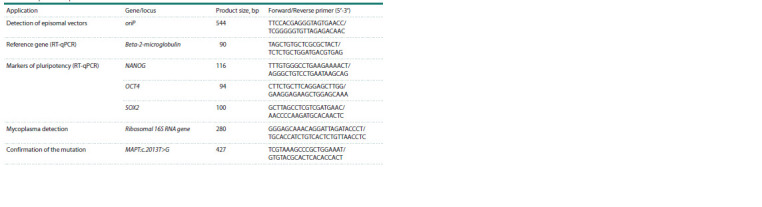
Sequences of primers used in the work

Quantitative RT-PCR for pluripotency markers. Reverse transcription of RNA was performed using M-MuLV Revertase (Biolabmix). Quantitative PCR was performed on a LightCycler 480 Real-Time PCR System (Roche) using the BioMaster HS-qPCR SYBR Blue 2× Kit (Biolabmix) according to the following programme: 95 °C – 5 min; 40 cycles: 95 °C – 10 s, 60 °C – 1 min. Primer sequences for pluripotency genes are shown in Table 2. CT values were normalised to beta-2-microglobulin using the ΔΔCT method.

PCR analysis for the detection of episomes and mycoplasma. Detection of mycoplasma contamination and presence of episome sequences in cells was performed by PCR (95 °C – 5 min; 35 cycles: 95 °C – 15 s, 60 °C – 15 s, 72 °C – 20 s) on a T100 Thermal Cycler (Bio-Rad) (Choppa et al., 1998; Okita et al., 2013). Primer sequences are listed in Table 2.

STR analysis. Genotyping of the studied DNA samples was performed at Genoanalytica LLC by polymerase chain reaction using a set of PCR reagents for direct amplification COrDIS EXPERT 26 (Russia) according to the manufacturer’s protocol, followed by separation of the amplification products on a capillary electrophoresis device 3130 Genetic Analyzer (HITACHI, Applied Biosystems Group of The Applera Corporation, Japan, registration certificate No. FSZ 2004/1586).

## Results and discussion

Analysing the results of exome sequencing of patients from the Federal Neurosurgical Centre (Novosibirsk) with a clinical diagnosis of Parkinson’s disease, a pathogenic genetic variant (c.2013T>G, rs63750756) in the MAPT gene associated with frontotemporal dementia with parkinsonism-17 was found in a 46-year-old patient. The first signs of parkinsonism in the patient were detected at the age of 44 years. In the family history, all female relatives had signs of the disease.

Mononuclear cells were isolated from the patient’s peripheral blood and reprogrammed by transfection with nonintegrating episomes expressing OCT4, SOX2, KLF4, LIN28, L-MYC and mp53DD (Okita et al., 2013). This resulted in ten lines being obtained. In the initial stages of the analysis, two lines were identified that met all the requirements for pluripotent stem cells. These lines (ICGi052-A/PD57-6 and ICGi052-B/PD57-7) have been characterized and registered in the Human Pluripotent Stem Cell Registry (hPSCreg, https:// hpscreg.eu). Full information on these lines is available in hPSCreg via the links https://hpscreg.eu/cell-line/ICGi052-A and https://hpscreg.eu/cell-line/ICGi052-B.

Both lines grow in dense monolayer colonies of cells with a high nuclear-cytoplasmic ratio and express an early marker of pluripotent cells, endogenous alkaline phosphatase (see the Figure, a). Cultivation was performed on mitotically inactivated mouse fibroblasts (or feeder). Immunofluorescence analysis of the ICGi052-A (passage 15) and ICGi052-B (passage 16) cell lines for pluripotency markers showed the expression of the surface antigens SSEA-4 and TRA-1-60 and the transcription factors NANOG and OCT4 (see the Figure, b). Quantitative real-time PCR (RT-qPCR) of the lines at passage 15 also showed a significant increase in the expression level of the OCT4, NANOG and SOX2 genes, comparable to the expression level in the control line of HUES9 embryonic stem cells (HVRDe009-A) (Cowan et al., 2004) (see the Figure, c). Karyotype analysis, involving 60 metaphase plates from each cell line, showed that cell lines ICGi052-A at passage 18 and ICGi052-B at passage 20 have normal diploid 46,XX karyotypes (see the Figure, d). In order to confirm the presence of a pathogenic polymorphism in the cell lines obtained, sequencing by the Sanger method was carried out and showed that both lines, as well as the patient’s PBMCs, carry the substitution c.2013T>G (see the Figure, e, SNPs are indicated by black arrows).

**Fig. 1.**
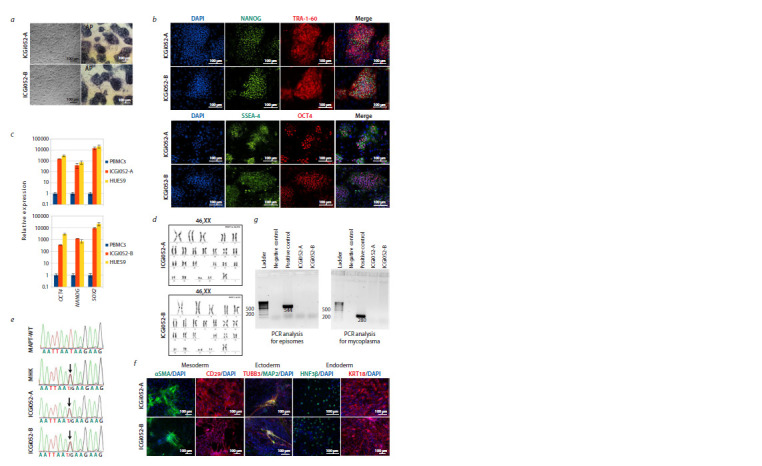
Characteristics of two patient-specific iPSC lines, ICGi052-A and ICGi052-B. a – morphology of cell colonies in phase contrast and histochemical detection of endogenous alkaline phosphatase (ALP) in iPSCs; b – immunofluorescence staining for pluripotency markers: NANOG (green signal), TRA-1-60 (red signal), SSEA-4 (green signal), OCT4 (red signal); c – real-time PCR for pluripotency markers (OCT4, SOX2, NANOG) of the ICGi052-A and ICGi052-B iPSC lines, patient PBMCs and HUES9 embryonic stem cell lines; d – karyotyping (DAPI-bending) of iPSC lines: ICGi052-A at passage 18 and ICGi052-B at passage 20; e – sequenograms of sections of the MAPT gene (c.2013T>G) PBMCs from a patient with frontotemporal dementia with parkinsonism-17, ICGi052-A and ICGi052-B iPSC lines and a healthy donor (MAPT-WT) (Sanger sequencing). The polymorphism is marked with a black arrow; f – immunofluorescence staining of spontaneously differentiated cells in embryoid bodies for markers of three germ layers: ectoderm (TUBB3 (red signal), MAP2 (green signal)), mesoderm (aSMA (green signal), CD29 (red signal)) and endoderm (keratin 18/KRT18 (red signal), HNF3β/FOXA2 (green signal)). The nuclei are stained with DAPI (4,6-diamino-2-phenylindole) (blue signal); g – the result of PCR analysis for episomes and mycoplasma contamination in the obtained cell lines. All scale bars are 100 μm.

The main test for the pluripotency of the cell lines obtained is their ability to differentiate into derivatives of three germ layers, for which spontaneous differentiation through embryoid bodies was performed, followed by immunofluorescence staining for specific markers. Both cell lines were shown to be capable of producing: ectoderm (tubulin β3 (TUBB3/ TUJ1), microtubule-associated protein 2 (MAP2)); endoderm (hepatocyte nuclear factor 3 beta (HNF3β/FOXA2), keratin 18 (KRT18)); mesoderm (smooth muscle α-actin (aSMA), surface marker CD29) (see the Figure, f ).

During cultivation, all lines were subjected to a PCR test for mycoplasma contamination, which confirmed its absence (see the Figure, g). It was also shown that the episomal vectors were eliminated by passage 19. STR analysis of short tandem repeats at 25 polymorphic loci at passages 15 (for ICGi052-A) and 16 (for ICGi052-B) showed the identity of patient-specific PBMCs (data available on request from the authors). The passport of the cell lines obtained is shown in Table 3.

**Table 3.**
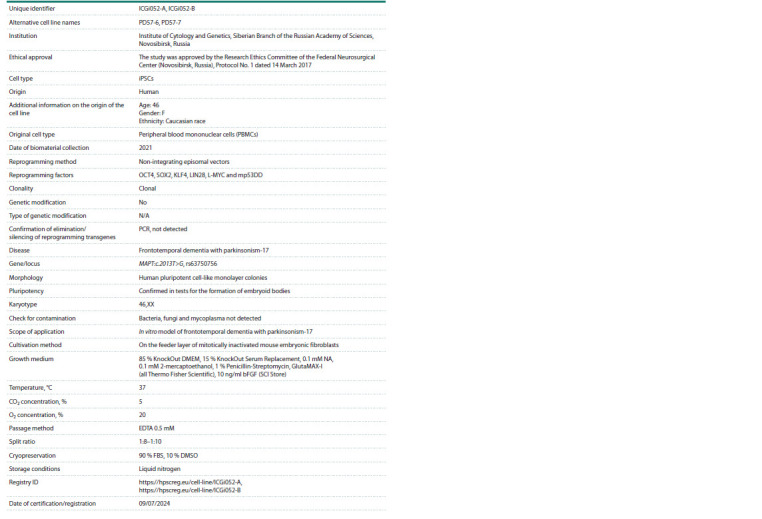
Passport of the ICGi052-A and ICGi052-B cell lines

## Conclusion

Using reprogramming into a pluripotent state, we have obtained and characterised in detail two lines of iPSCs – ICGi052-A and ICGi052-B – from the PBMCs of a patient with frontotemporal dementia with parkinsonism-17, who has a pathological genetic variant c.2013T>G (rs63750756) in the MAPT gene. These cell lines meet all the criteria of pluripotent cells (they have a diploid karyotype, express pluripotency markers and are capable of producing derivatives of three germ layers) and are a unique tool for the in vitro study of the development of pathology in neural derivatives obtained by directed differentiation of iPSCs, as well as for testing new pharmacological compounds. The cell lines obtained are registered in the Human Pluripotent Stem Cell Registry of hPSCreg and are also included in the cell collection of the Laboratory of Developmental Epigenetics of IC&G SB RAS and can be used to study the pathological mechanisms of the progression of taupathies.

## Conflict of interest

The authors declare no conflict of interest.
